# Osteoclasts and Microgravity

**DOI:** 10.3390/life10090207

**Published:** 2020-09-16

**Authors:** John Kelly Smith

**Affiliations:** Departments of Academic Affairs and Biomedical Sciences, James H Quillen College of Medicine, East Tennessee State University, P.O. Box 70300, Johnson City, TN 37614, USA; smithj@etsu.edu; Tel.: +1-423-794-7425

**Keywords:** osteoclasts, microgravity, spaceflight, osteoblasts, osteocytes, M-CSF, RANKL, bone, microgravity, cytokines

## Abstract

Astronauts are at risk of losing 1.0% to 1.5% of their bone mass for every month they spend in space despite their adherence to diets and exercise regimens designed to protect their musculoskeletal systems. This loss is the result of microgravity-related impairment of osteocyte and osteoblast function and the consequent upregulation of osteoclast-mediated bone resorption. This review describes the ontogeny of osteoclast hematopoietic stem cells and the contributions macrophage colony stimulating factor, receptor activator of the nuclear factor-kappa B ligand, and the calcineurin pathways make in osteoclast differentiation and provides details of bone formation, the osteoclast cytoskeleton, the immune regulation of osteoclasts, and osteoclast mechanotransduction on Earth, in space, and under conditions of simulated microgravity. The article discusses the need to better understand how osteoclasts are able to function in zero gravity and reviews current and prospective therapies that may be used to treat osteoclast-mediated bone disease.

## 1. Introduction

The skeletal system of vertebrates has had millions of years to adapt to the force of gravity on Earth (9.8 m/s^2^) and to allow osteocytes and the immune system to balance the activities of osteoblasts and osteoclasts. This osteoimmunological system is complex and involves commonly shared osteoclastogenic factors such as receptor activator of the nuclear factor-kappa B (NF-κB) ligand and macrophage colony stimulating factor as well as cytokines and immune cells that inhibit or enhance osteoclast ontogeny [1]. This adaptation has involved the construction of cytoskeletons supported by actin and intermediate filaments and microtubules [2,3], intracellular adhesion molecules including integrins and cell extension kinases [4,5], plasma membrane and nuclear mechanosensors [6], and thermal convection currents which renew nutrients and remove waste [7].

Man’s venture into the vacuum of space where the force of gravity is one millionth of that on Earth has resulted in adverse effects on the osteoimmunological system, particularly bone homeostasis [8]. Astronauts are not only at risk of progressive bone and cartilage loss while in space but must also face the reality that space-related bone and joint changes may persist for years after their return to Earth despite efforts made to protect their skeletal systems [8,9,10,11,12,13].

In this review, I describe the ontogeny of osteoclast hematopoietic stem cells and the contributions macrophage colony stimulating factor, receptor activator of NF-κB ligand, and the calcineurin pathways make in osteoclast differentiation and provide details of bone formation, the osteoclast cytoskeleton, the immune regulation of osteoclasts, and osteoclast mechanotransduction on Earth, in the microgravity of space, and in conditions of simulated microgravity. The article discusses the need to better understand how osteoclasts are able to function in zero gravity and reviews current and prospective therapies that may be used to treat osteoclast-mediated bone disease.

## 2. Materials and Methods

This narrative review is on the effect of microgravity on osteoclast ontogeny and function. The research strategy included the following: 1. defining the key topics; 2. identifying key words or synonyms that represent each of the key topics; 3. an online PubMed search of key topics and key words; and 4. a refinement of the search based on initial findings. Data restrictions included articles with identical samples and identical outcomes, identical samples with different outcomes, increased samples and identical outcomes, and decreased samples with identical outcomes. Key topics included bone homeostasis, osteoclast stem cells, osteoclast differentiation, osteoclast cytoskeleton, osteoclast immunoregulation, osteoclast mechanosensors, osteoclast mechanotransduction, osteoclasts and microgravity, osteoclasts and simulated microgravity, osteoclast-mediated bone disease and treatment, osteocytes, osteoblasts, osteoblast stem cells. Keywords include hematopoietic stem cells, receptor activator of nuclear factor-kappa B, receptor activator of nuclear factor-kappa B ligand, macrophage colony stimulating factor, osteoprotegerin, calcineurin, stromal-cell-derived factor-1, sphingosine-1-phosphate, tumor necrosis factor-α, interleukins, interferon-γ, sclerostin, bisphosphonates, denosumab, odanacatib, romosozumab, melatonin, insulin-like growth factor-1, bone morphogenetic proteins, and transforming growth factor-β. 

## 3. Bone Homeostasis

**Figure 1:**
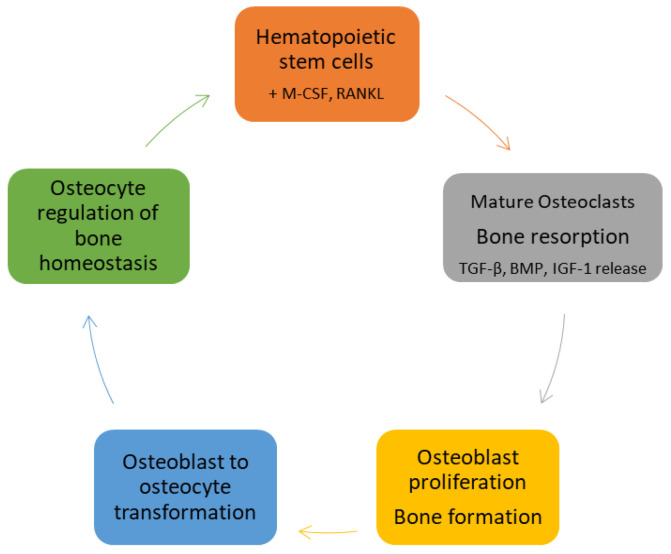
The cycle of bone formation. Hematopoietic stem cell ligation of macrophage colony stimulating factor (M-CSF) and receptor activator of NF-κB ligand (RANKL) initiates their differentiation into mature bone resorbing osteoclasts. Osteoclast-mediated bone resorption releases osteoblast growth factors transforming growth factor (TGF)-β, bone morphogenetic proteins (BMPs), and insulin-like growth factor (IGF)-1 resulting in osteoblast differentiation from mesenchymal stem cells, with subsequent bone formation and mineralization. Osteoblasts trapped in bone matrix evolve into osteocytes, the most abundant cells in bone and the primary regulators of bone homeostasis.

Osteoclast precursors are recruited to bone remodeling units (BRUs) by gradients of stromal-cell-derived factor-1 (SDF-1). At BRUs they differentiate into mature multinucleated osteoclasts by binding macrophage colony stimulating factor (M-CSF) and receptor activator of NF-κB ligand (RANKL) secreted by resident stromal cells, osteocytes and osteoblasts. The newly formed osteoclasts secrete hydrochloric acid, cathepsin K, and matrix metallopeptidase (MMP)-9, degrading cortical and trabecular bone and releasing embedded growth factors including bone metamorphic proteins (BMPs), transforming growth factor (TGF)-β, and insulin-like growth factor (IGF)-1. Once resorptive pits reach a given size, osteoclasts undergo apoptosis, terminating bone resorption. The newly liberated growth factors stimulate osteoblastogenesis, increasing the numbers of mature osteoblasts available to form and mineralize new bone. Eventually, osteoblasts are trapped in the bone matrix where they evolve into osteocytes. Osteocytes comprise about 95% of bone cells. They reside in lacunae and communicate with other osteocytes, osteoblasts, and osteoclasts via dendritic processes that traverse a series of canals in the bone matrix (the lacunar–canalicular network). Osteocytes respond to hydrostatic and fluid shear stresses in the lacunae and canaliculi and are largely responsible for the control of bone homeostasis [8] (Figure 1).

## 4. Osteoclast Stem Cells

### 4.1. Osteoclast Stem Cell Niche

Goto and associates have provided evidence that a small population of cysteine x cysteine receptor 4^+^ cluster of differentiation 45^−^ (CXCR4^+^ CD45^−^) bone marrow cells provide a niche for osteoclastogenesis. These cells express low levels of receptor activator of NF-κB (RANK) and its ligand RANKL but high levels of essential chemokines including SDF-1, chemokine (C-X-C motif) ligand 7 (CXCL7), and chemokine (C-X3-C motif) ligand 1 (CX3CL1). Their findings suggest that CXCR4^+^ CD45^−^ cells support an appropriate microenvironment for osteoclastogenesis, with a direct effect on cells expressing SDF-1, CXCL7, and CX3CL1 receptors [14].

### 4.2. Osteoclast Stem Cell Circulation

**Figure 2:**
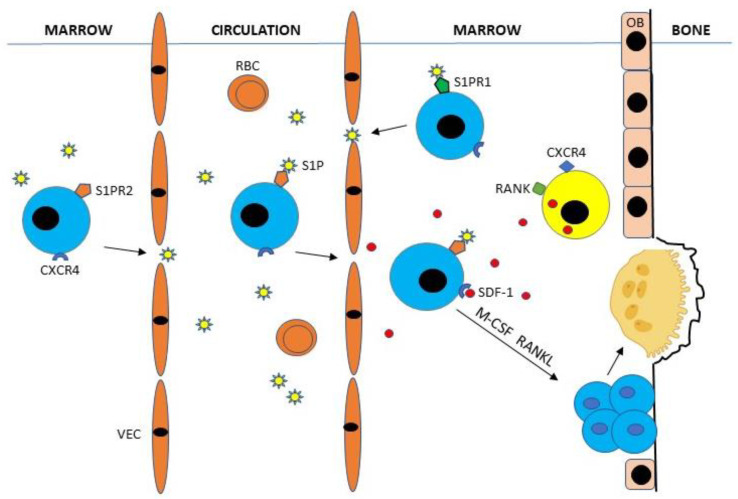
Bone marrow hematopoietic stem cells (HSCs) (colored blue) expressing type-2 receptor for sphingosine-1-phosphate (S1PR2) enter the circulation by binding S1P, a chemotactic lysophospholipid normally present in high concentrations in blood. Circulating CXCR4-expressing HSCs are attracted to bone surfaces by gradients of stromal-cell-derived factor-1 (SDF-1) (CXCL12) secreted by CXCR4^+^ RANK^+^ CD45^−^ stromal marrow cells (colored yellow). HSCs may then be recycled to the bone marrow by binding S1P to S1PR1 or stay at bone surfaces where they evolve into mature, bone-resorbing osteoclasts by binding M-CSF and RANKL produced primarily by osteoblasts and osteocytes. OB, osteoblast; RBC, red blood cell; VEC, vascular endothelial cell.

Bone marrow osteoclast hematopoietic stem cells (HSCs) expressing type-2 receptor for sphingosine-1-phosphate (S1PR2) enter the circulation by binding sphingosine-1-phosphate (S1P), a chemotactic lysophospholipid normally present in high concentrations in blood [15,16]. Circulating CXCR4-expressing HSCs are attracted to bone surfaces by gradients of SDF-1 (CXCL12) secreted by CXCR4^+^ CD45^−^, stromal, and endothelial marrow cells [14,17]. HSCs may then be recycled to the bone marrow by binding S1P to type-1 receptors (S1PR1) or stay at bone surfaces where they evolve into mature osteoclasts by binding M-CSF and RANKL produced primarily by osteoblasts, osteocytes, and stromal cells [15,16] (Figure 2).

## 5. Osteoclast Differentiation

### 5.1. M-CSF Pathway

The differentiation of HSCs into osteoclast precursors requires the expression of spleen focus-forming virus proviral integrin 1 (PU.1), heterodimeric complex of microphthalmia-associated transcription factor (MITF), and transcription factor E3 (TfE3), which initiate the expression of the colony stimulating factor-1 receptor (C-Fms) [18]. Ligation of M-CSF to C-Fms initiates the expression of RANK, the cognate receptor for RANKL. M-CSF ligation also initiates the expression of several cytokines and their receptors including interleukin (IL)-1α, IL-18, interferon (IFN)-β, IL-11Rα2, IL-6/11R gp130, and IFN-γ-R; also expressed are factors involved in the cell’s response to RANKL ligation, including tumor necrosis factor receptor-associated factor 2A (TRAF2A), phosphatidylinositol 3-kinase (PI3K), mitogen-activated protein kinase kinase kinase 3 (MEKK3), and receptor-interacting serine-threonine kinase 1 (RIPK1) [19].

### 5.2. RANKL Pathway

**Figure 3:**
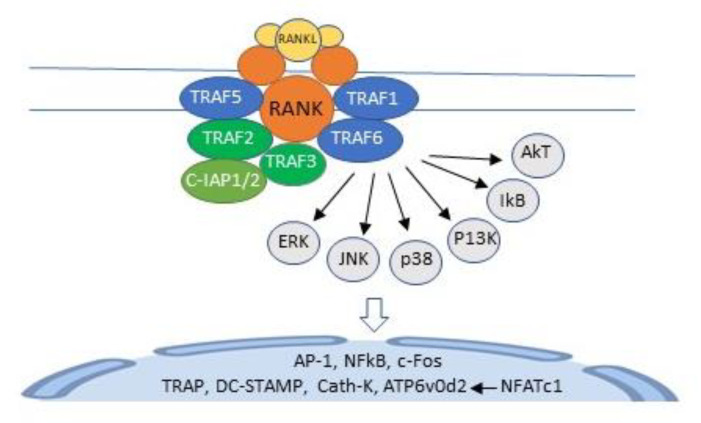
Binding of RANKL to RANK activates TNF receptor activating factors (TRAFs) 1, 2, 3, 5, and 6. TRAF 6 recruits and activates a kinase cascade, which includes extracellular regulated kinase (ERK), c-jun, N-terminal kinase (JNK), p38 mitogen-activated protein kinase (p38), phosphatidylinositol-3 kinase (PI3K), IkB, and AkT. This cascade initiates the transcription of AP-1, c-Fos, NF-κB, and nuclear factor of activated T cells cytoplasmic calcineurin-dependent 1 (NFATc1), with consequent induction of the osteoclast specific genes, *TRAP, cathepsin K, DC-STAMP, and ATP6v0d2.* C-IAP1/2, cellular amino-terminal inhibitor of apoptosis 1 and cellular amino-terminal inhibitor of apoptosis 2; AKT, a serine protein kinase from the protein kinase AGC subfamily;

Ligation of RANKL to its cognate receptor RANK activates TNF receptor-activating factors (TRAFs) 1, 2, 3, 5, and 6, adapter proteins that recruit and activate protein kinases. In unstimulated HSCs, TRAFs 2 and 3, and c baculoviral inhibitor of apoptosis repeat-containing protein 2 (cIAP1) form a complex that polyubiquitinates NF-κB-inducing kinase (NIK), which is transported to the proteasome for degradation, resulting in very low levels of NF-κB in unstimulated HSCs [18]. In RANKL-activated HSCs, polyubiquitination of NIK is inhibited [18], and TRAF6 activates a cascade of kinases, including extracellular regulated kinase (ERK), p38 mitogen-activated protein kinase (p38), c-jun N-terminal kinase (JNK), phosphatidylinositol-3 kinase (PI3K), and Akt and IkB kinases [20,21]. This cascade requires Lys63-linked TRAF-6 auto-ubiquitination [22] and initiates the transcription of activator protein-1 (AP-1), c-Fos, NF-κB, and nuclear factor of activated T cells cytoplasmic calcineurin-dependent 1 (NFATc1), the master regulator of osteoclast differentiation [20,23,24]. NFATc1 binds to its own promoter, switching on an epigenetically controlled autoregulatory cycle that permits efficient induction of the osteoclast-specific genes, *tartrate-resistant acid phosphatase (TRAP), cathepsin K,* as well as the fusion-specific genes, *dendritic cell-specific transmembrane protein (DC-STAMP) and ATPase H^+^ transporting V0 subunit D2 (ATP6v0d2*) [20,25,26] (Figure 3).

### 5.3. Calcineurin Pathway

**Figure 4:**
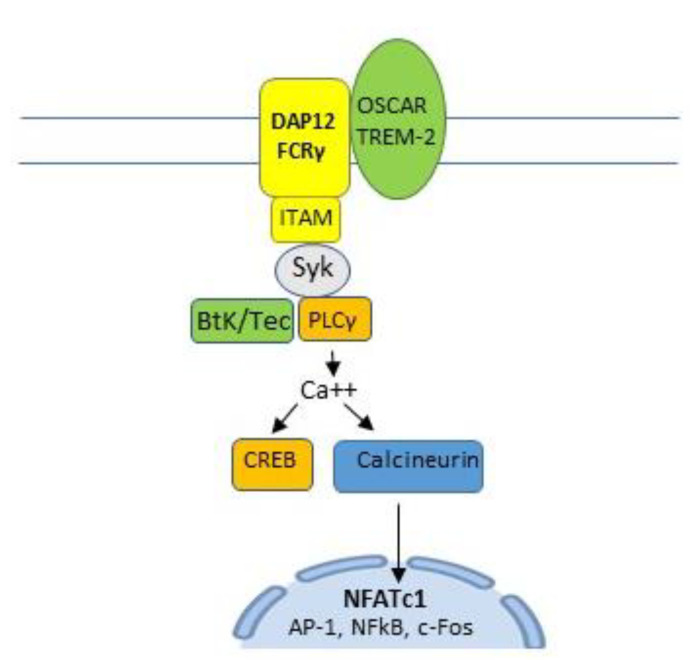
Membrane expressed osteoclast-associated receptor (OSCAR) and triggering receptor expressed in myeloid cells-2 (TREM2) pair with adaptor molecules, Fc receptor common gamma chain (FcRγ) and DNAX-activating protein 12 k-DA (DAP12) to activate immunoreceptor tyrosine-based activation motif (ITAM). ITAM activates spleen tyrosine kinase (SyK), Burton’s tyrosine kinase (BtK), and phospholipase C gamma (PLCγ) to induce calcium signaling which is required to activate cAMP response element-binding protein (CREB) and the calcineurin pathway, a key costimulatory pathway of NFATc1 and an important signaling component of a number of immune cell receptors.

**Figure 5:**
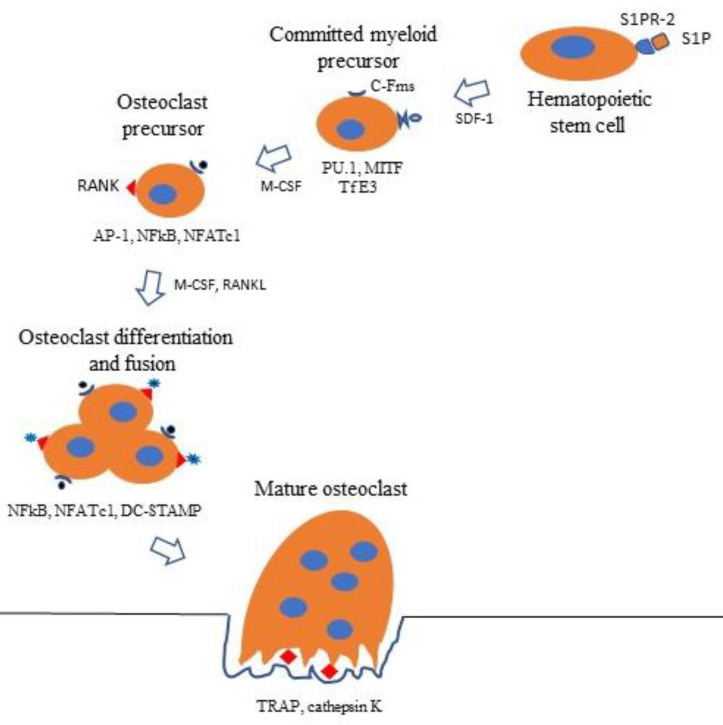
Circulating HSCs binding S1P to S1PR2 are attracted to bone surfaces by chemokines such as SDF-1. Here, they differentiate into committed myeloid precursors expressing proviral integrin 1 (PU.1), microphthalmia-associated transcription factor (MITF), and transcription factor E3 (TfE3), transcription factors that induce the expression of C-Fms, the receptor for M-CSF. Binding of M-CSF to C-Fms results in the expression of transcription factors activator protein 1 (AP-1), NF-κB, NFATc1, and RANK, the receptor for RANKL. Binding of M-CSF and RANKL to their cognate receptors promotes further differentiation into osteoclast precursors expressing transcription factors NF-κB, NFATc1, and *dendritic cell-specific transmembrane protein (DC-STAMP).* These cells fuse, forming mature *TRAP-, cathepsin K*-positive osteoclasts.

Membrane-expressed osteoclast-associated receptor (OSCAR) and triggering receptor expressed in myeloid cells-2 (TREM2) pair with adaptor molecules, Fc receptor common gamma chain (FcRγ) and DNAX-activating protein 12 kDa (DAP12), to activate immunoreceptor tyrosine-based activation motif (ITAM). ITAM activates spleen tyrosine kinase (SyK) which, in turn, activates Bruton’s tyrosine kinase (BtK) and phospholipase C gamma (PLCγ) to induce calcium signaling. This activates cyclic adenosine monophosphate (cAMP) response element-binding protein (CREB) and the calcineurin pathway [21,24,25,27], a key costimulatory pathway of NFATc1 [21,23] and an important signaling component of a number of immune cell receptors [28] (Figure 4 and Figure 5).

## 6. Osteoclast Cytoskeleton

### 6.1. Cytoskeleton Elements

The osteoclast cytoskeleton is made of filamentous structures that belong to one of four categories: polarized actin filaments; polarized microtubules; non-polarized intermediate filaments; or non-polarized septin filaments. The cytoskeleton fulfills essential functions including cell adhesion, migration, contractility, division, vesicular transport, and bone resorption [2,3,4,5,6].

#### 6.1.1. The Sealing Zone

**Figure 6:**
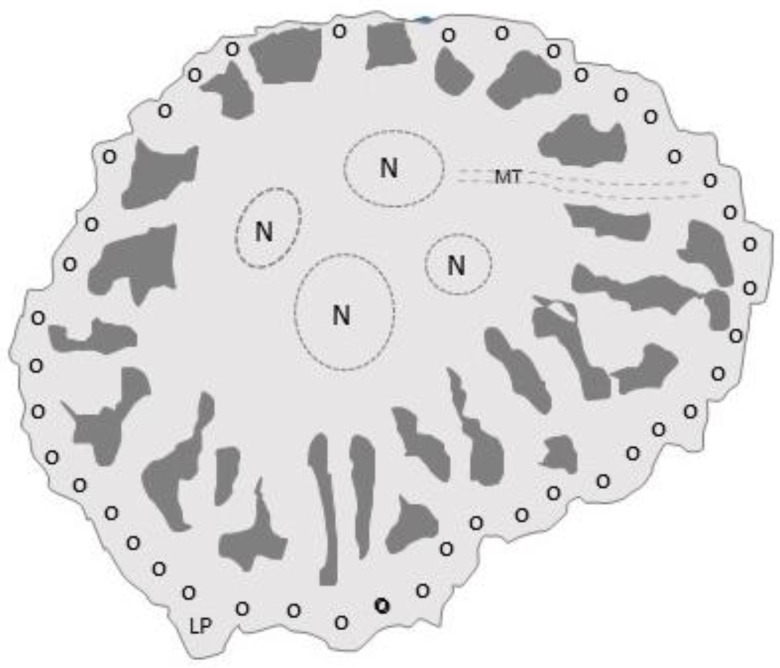
Top view tracing of a photomicrograph of an adherent osteoclast showing the sealing zone’s podosomes (small circles). Microtubules (dotted lines) extend from perinuclear regions to the podosome belt. When they reach the belt, they can stop, bend back toward the cell center, cross the belt, or form a circular network above the belt. Hydrochloric acid and proteases are secreted inside the sealing zone to resorb bone. The typical lifespan of an osteocyte is two weeks. N, nucleus; MT, microtubules; LP, lamellipodia.

Osteoclasts adhere to bone by means of the sealing zone, a belt of densely packed microtubule-stabilized podosomes (Figure 6). Podosomes mediate adhesion via integrin αvβ3, the major osteoclast integrin. In the center of the sealing zone, the osteoclast membrane differentiates into a ruffled border, where it secretes hydrochloric acid and proteases (cathepsin-K, MMP9, and MMP14) to dissolve hydroxyapatite crystals and bone matrix, respectively [2].

#### 6.1.2. The Actin Cytoskeleton

The core domain of podosomes consists of branched actin that polymerizes below the plasma membrane. The core is surrounded by unbranched actinomysin filaments, which connect to integrins and link neighboring podosomes. The core also contains adhesion protein CD44. Although the typical half-life of a podosomes is measured in minutes, the podosome belt is made of thousands of podosomes and can last for hours [2].

The tyrosine kinase, Src, is a key controller of podosome dynamics and organization. Src binds to tyrosine kinase Pyk2 resulting in their activation and regulation of podosome dynamics largely through small GTPases of the Rho family [2].

#### 6.1.3. Crosstalk between Actin and Microtubular Networks

The actin motor protein, unconventional myosin X (Myo10), can bind actin, microtubules, and integrins, and has been proposed to crosslink actin cytoskeleton and microtubules in osteoclasts [2].

#### 6.1.4. Intermediate and Septin Filaments

Intermediate filaments include vimentin, plectin, and fimbrin; they connect the nuclear and plasma membranes with microtubules and actin filaments. Vimentin filaments are found along microtubules and the podosomal belt, and plectin and fimbrin are both podosomal proteins. Plectin is required for microtubule acetylation and Src and Pyk2 activities, and, along with fimbrin, connects vimentin to actin filaments [2].

Relatively little is known about the functions of the 13 septin filaments. Septin 9 links septin filaments to other cytoskeletal elements and membranes, bundles microtubules, and inhibits the activity of myosin and cofilin. It is associated with actin filaments and microtubules in the sealing zone, and its inhibition is detrimental to bone resorption [2].

## 7. Immunoregulation

### 7.1. Cytokines

Osteoclasts are immunologically reactive cells that have taken over the duties of balancing bone resorption with bone formation. As such, they are responsive to a variety of pro-inflammatory cytokines, which generally increase their resorptive capacity, and anti-inflammatory cytokines, which generally diminish their ability to resorb bone. These interactions play a critical role in inflammatory bone and joint diseases such as rheumatoid arthritis, ankylosing spondylitis, psoriatic arthritis, and osteoarthritis, particularly post-traumatic osteoarthritis. The following lists cytokines based on whether their predominant effect on osteoclastogenesis is favorable or inhibitory.

#### 7.1.1. Osteoclastogenic Cytokines

Foremost among the osteoclastogenic cytokines is TNF-α, a potent stimulator of osteoclastogenesis and a dominant cytokine in most bone and joint inflammatory diseases including post-traumatic osteoarthritis. In this regard, it is important to note that astronauts experience traumatic joint injuries involving their knees, ankles, hips, and shoulders at almost three times the rate as individuals on Earth.

TNF-α induces osteoclastogenesis in RANKL- and M-CSF-positive hematopoietic cell precursors [29,30], where its effects are mediated by TNF-α receptors type 1 (p55r) and type 2 (p75r), the former being the most effective in inducing the differentiation of TRAP-positive multinucleated cells [31]. In addition, TNF-α has the capacity to inhibit osteoblastogenesis by downregulating the expression of IGF-1 in mesenchymal stem cell precursors [32].

Also prominent among the osteoclastogenic cytokines are IL-1α and IL-1β. Tanabe and associates examined the effect of IL-1α on cultures of rat osteoblasts and hematopoietic stem cells and found that it stimulated osteoclastogenesis by upregulating M-CSF and prostaglandin E2 (PGE2) production and by decreasing OPG production in osteoblasts [33]. Azuma and associates found that IL-1β enhanced the ability of TNF-α to upregulate bone resorption by TRAP-positive multinucleated cells [31]. However, IL-1β has also been reported to suppress osteoclast formation by upregulating OPG production by chondrocytes [34].

Transforming growth factor-β, which is released from the bone matrix during bone resorption, has been shown to enhance osteoclast differentiation in RANKL- and M-CSF-stimulated HSC cultures [35]. Bone morphogenetic protein-1 in conjunction with RANKL has been shown to increase the differentiation and survival of osteoclasts [36]. Moreover, IL-7 + RANKL produced by activated T cells is reported to stimulate osteoclastogenesis in cultures of peripheral blood mononuclear cells [37]. IL-34 produced by osteoblasts recognizes the receptor for M-CSF (C-Fms) on osteoclast progenitors, thereby promoting osteoclastogenesis [38]. In association with inflammatory joint diseases such as rheumatoid arthritis, IL-17 produced by T17 lymphocytes serves as a potent osteoclastogenic cytokine [1].

#### 7.1.2. Anti-Osteoclastogenic Cytokines

Foremost among the anti-osteoclastogenic cytokines are IL-4 and IL-10. Mohamed and associates found that low doses of IL-4 inhibited RANKL-induced osteoclastogenesis in monocytes/macrophages and in bone marrow osteoclast precursors by downregulating NFATc1 mRNA expression [39]. In keeping with these findings, Wei and associates found that IL-4 inhibited osteoclastogenesis by blocking the JNK, p38, and ERK protein kinase pathways, which are upstream of NFATc1 [40]. In their study on the effect of IL-4 on TNF-α-mediated osteoclast formation in live murine calvariae, Fujii and associates found that IL-4 also inhibited RANKL expression in TNF-α-activated stromal cells [41]. Furthermore, Zhao and Ivashkiv reported that IL-4 limits bone resorption by promoting OPG expression and suppressing the expression of RANKL, RANK, NF-κB, c-Fos, NFATc1, MAPK, and calcium signaling during osteoclast formation [42]. Using a RAW267.4 macrophage cell line and murine bone marrow cells, Mohamed and associates reported that IL-10 inhibited osteoclastogenesis by downregulating RANKL-induced expression of c-Fos and its downstream target, NFATc1 [43]. Additionally, Zhao and Ivashkiv reported that IL-10 limits osteoclast formation by inhibiting the expression of c-Fos, c-Jun, TREM2, and NFATc1 in osteoclast precursors [42].

IL-6, IL-12, IL-18, and interferon (IFN)-γ also possess anti-osteoclastogenic properties. Honda studied cultures of chondrocytes containing IL-6 and its soluble receptor, sIL-6r, for up to 28 days; he then tested the culture supernatant for its ability to induce differentiation of RAW264.7 cells into osteoclast precursors. He found that IL-6 and IL-6r suppressed the differentiation of osteoclasts by inducing chondrocytic PGE2 [44]. Using mouse bone marrow cultures, Kitaura and associates found that IL-12 when added to TNF-α-stimulated cultures induced osteoclast apoptosis by upregulating their expression of Fas/Fas ligands [45]. Using injections of TNF-α alone and with IL-18 or IL-18 + IL-12 into live murine supracalvaria, Morita and associates found that IL-12 enhanced IL-18s ability to inhibit TNF-α-mediated osteoclastogenesis and that the inhibition was T-cell independent [46]. Horwood and associates also reported that IL-12 inhibited osteoclast formation in vitro [47]. Using co-cultures of osteoblasts and hematopoietic cells, Udagawa and associates found that IL-18 inhibited osteoclastogenesis by downregulating osteoblast production of M-CSF [48]. Moreover, using cultures of murine bone marrow macrophages, Kohara and associates found that IFN-γ directly inhibited TNF-α-mediated osteoclastogenesis and induced osteoclast precursor apoptosis by upregulating Fas/Fas ligand binding [49] (Table 1).

## 8. Mechanotransduction

Cellular responses to external forces are mediated by actin, microfilaments, and microtubules of the cytoskeleton, by integrins and other intracellular adhesion molecules, and by membrane and nuclear mechanosensors. Cell extension kinases, gravisensing organelles, and thermal convection currents that enable heat and nutrient exchange and removal of waste are also involved [1,2,3,4,5,6,7]. In addition to the force of gravity, mechanotransduction in bone is mediated by fluid shear stresses [50], hydrostatic pressures [51], and the force of muscular contraction [52].

Osteoclast responses to mechanical loading are mediated largely by osteoblasts and osteocytes. Osteoblasts inhibit osteoclastogenesis by reducing the available amounts of RANKL by producing OPG, a decoy receptor for RANKL. Osteocytes control osteoclast activity by secreting sclerostin, which increases bone resorption by downregulating osteoblast OPG production and decreases osteoblastogenesis by inhibiting Wnt/β-catenin signaling pathways in osteoblast precursors [53].

### 8.1. Mechanotransduction in Space (10^−6^ g)

The force of gravity in space is one millionth of that experienced on Earth (9.8 m/s^2^). In stark contrast to the devastating effects of microgravity on osteoblasts, whose cytoskeletons may implode do to the failure of microtubules and actin filaments [54,55], and osteocytes, which undergo apoptosis as early as three days into space flight [56], osteoclasts maintain their ability to resorb bone in microgravity. Along with the diminished force of muscular contraction on bone, these changes account for the space-related loss of bone mass. Furthermore, the detrimental effects of space travel on bone and cartilage can persist for years after astronauts return to Earth.

#### 8.1.1. Studies Using Live Animals

Early studies on the effects of spaceflight on bone homeostasis were reported by Vico and associates. In one study, they flew seven Wistar rats for seven days aboard BIOCOSMOS 1667 and performed histomorphometric studies on the tibias, femurs, and thoracic and lumbar vertebrae of the sacrificed animals. Microgravity reduced tibial trabecular bone volume by 47–55%, trabecular thickness by 20–24%, and trabecular density by 40–43% as compared to ground controls. In contrast, femoral trabecular bone at the site of muscular insertions and vertebral bone showed no significant changes when compared to ground controls. The authors were unable to explain the different outcomes for the weight-bearing bone models [57]. Using similar methods, the same investigators had previously shown that 5 days of spaceflight aboard the COSMOS 1514 biologic satellite increased the number of TRAP-positive osteoclasts per mm^2^ of thoracic and lumbar vertebrae [58] and that 14 days of flight aboard the COSMOS 2044 space shuttle increased the osteoclast number and resorptive activity in the male Wistar rat tibial metaphysis [59].

Blaber and associates analyzed the pelvic and femoral bones of eight C57BL/6 mice flown for 15 days on the STS-131 space shuttle mission using nano-CT scans and measurements of TRAP. They found that microgravity reduced the bone volume by 6.3% and bone thickness by 11.9% and increased the number of bone-surface TRAP-positive osteoclasts by 170% compared to that of ground controls, indicating enhanced osteoclast-mediated bone resorption. They also documented osteocyte apoptosis as reflected by increases in lacunar and canalicular diameters [60].

Chatani and associates used medaka fish and medaka fish larvae to study osteoclast function during two flights aboard the International Space Shuttle. They used double transgenic fish with a TRAP promoter linked to a green fluorescent protein and a metalloproteinase 9 (MMP9) promoter linked to a red fluorescent protein to identify osteoclasts. The fish were studied in vivo using a remote-controlled microscope on board the Space Shuttle; RNA was extracted from several fish on days 4 and 6 of spaceflight for subsequent transcriptome analysis. They found that in vivo signals of osteoclast activity were upregulated at days 4 and 6 after launch, and that expression of osteoclast-specific genes TRAP, cathepsin K, and MMP9 were upregulated on day two of the flight [61].

#### 8.1.2. Studies Using Cell Cultures

Nabavi and associates measured the effects of 5 days of spaceflight on cultures of mouse calvaria osteoblasts and osteoclasts derived from RANKL-stimulated RAW 264.7 murine macrophage cell lines. To measure their resorptive capacity, osteoclasts were subcultured on Osteologic CC2 slides and Narwal ivory slices prior to flight. On return from space, osteoblasts were immunostained with monoclonal antibodies to α-tubulin, acetylated tubulin, vinculin, paxillin, zyxin, and tubulin and examined by fluorescent microscopy. The authors found that spaceflight osteoblasts had shorter and curvier microtubules, a reduced number and size of focal adhesins, and more condensed and fragmented nuclei when compared to ground controls, findings in keeping with other reports on the adverse effects of microgravity osteoblast cytoskeleton integrity. In contrast, on analyzing approximately 16,000 resorptive pits on Osteologic slides, they found that microgravity increased the resorptive activity of osteoclasts when compared to that of ground controls, underscoring the resilience of these cells to the effects of zero gravity [54].

Tamma and associates flew bioreactor cultures of osteoclasts and their precursors for 10 days on the FOTON-M3 mission. Osteoclast precursors were cultured on skelite (a 3-dimensional bone-like material) or devitalized bovine bone. They found that microgravity increased by several-fold the expression of genes involved in osteoclast activation and function; osteoclast bone resorption was also increased as evidenced by increases in collagen telopeptide production as compared to that of ground controls [62].

#### 8.1.3. Summary

**Figure 7:**
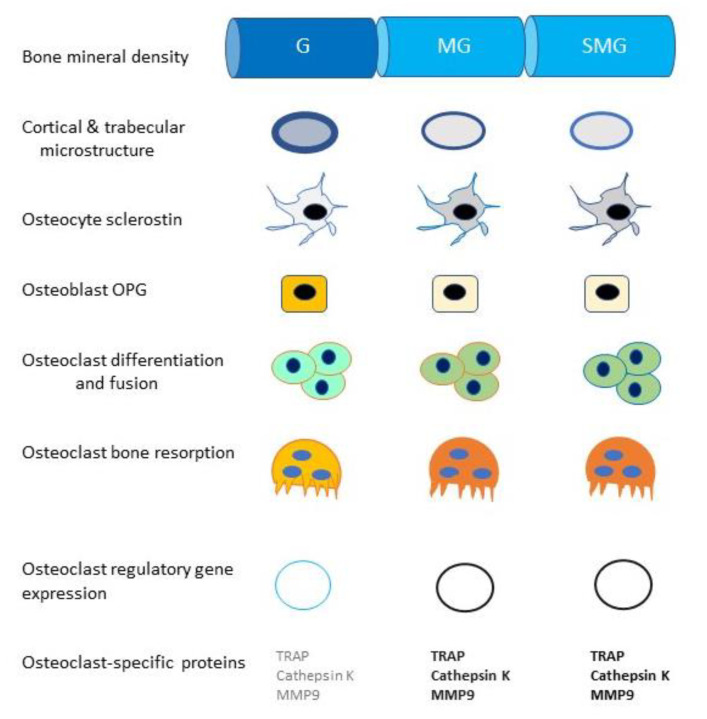
Comparison of bone homeostasis in Earth’s gravity (G), in the microgravity of space (MG), and under conditions of simulated microgravity (SMG). The darker colors and thicker lines indicate increased bone structure or enhanced activity. Bone mineral densities and cortical and trabecular microstructures are decreased and osteocyte secretion of sclerostin is increased in MG and SMG. Sclerostin inhibits osteoblastogenesis and bone formation by blocking Wnt/β-catenin signaling in osteoblast stem cells and enhances osteoclastogenesis by reducing osteoblast production of RANKL-binding osteoprotegerin (OPG). Osteoclast differentiation and fusion, bone resorption, expression of regulatory genes, and production of osteoclast-specific proteins are also upregulated in MG and SMG.

Both live animal and cell culture studies are uniform in documenting an upregulation of osteoclast numbers, resorptive activity, and resorption-related gene expression in the microgravity of space (bone volumes, masses) (Figure 7).

### 8.2. Mechanotransduction in Simulated Microgravity (g ≥ 10^−3^)

#### 8.2.1. Studies Using Live Animals

Saxena and associates subjected 6–7-week-old male wild-type BALB/c mice to 4 weeks of 30° head-down hindlimb unloading and then performed histomorphometric studies and measured femoral bone mineral density by dual-energy X-ray absorptiometry (DXA) and femoral trabecular and cortical bone integrity by micro-CT scanning. Ex vivo cell analysis was done on tibial bone marrow cells differentiated by the addition of RANKL and M-CSF; at 4 days, RNA extracts and DNAc were prepared. They found that hindlimb unloading caused a decrease in femoral bone mineral density and cortical and trabecular bone microstructure. This loss was associated with an upregulation in osteoclastogenesis-related signaling molecules ERK, p38, PLCγ2, and NFATc1 and osteoclast-specific markers TRAP and cathepsin K. They concluded that modeled microgravity increases osteoclast precursor differentiation with consequent increases in bone resorption [63].

Aguirre and associates subjected Swiss Webster mice to 1–18 days of 30° head-down tail suspension and performed histomorphometric, bone density DXA analysis, and bone compression resistance studies on lumbar vertebrae at various time intervals. They found evidence of osteocyte apoptosis on day 3 of suspension followed by increased endosteal levels of osteoclast numbers and resorptive activity and decreased bone density and compressive resistance at 18 days of tail suspension. They concluded that simulated gravity causes osteocyte apoptosis and consequent increases in levels of osteoclasts and osteoclast-mediated bone resorption [64] (Figure 7).

#### 8.2.2. Studies Using Cell Cultures

Rucci and associates studied the effect of simulated microgravity on osteoclastogenesis and bone resorption using 7-day-old CD1 mice calvariae cells for osteoblast cultures and CD1 mouse long bone marrow cells for osteoclast cultures. Cultures were done using NASA’s Rotating Wall Vessel Bioreactors. Osteoblasts were cultured for 24 h and analyzed for the expression of alkaline phosphatase (Alp), runt-related transcription factor-1 (Runx2), parathyroid hormone (PTH)/PTH peptide (P) receptor, and type I collagen and osteocalcin (OCN); osteoblast culture supernatants were assayed for osteoprotegerin (OPG) and RANKL concentrations and used as media for osteoclast cultures. Osteoclasts were cultured for 7 days on bone slicers and in the osteoblast culture media, stained for TRAP, and assayed for resorptive activity by measuring the number of bone slice resorption pits. The investigators found that simulated microgravity enhanced osteoclastogenesis by decreasing osteoblast production of OPG (increasing RANKL/OPG ratios) [65].

Using NASA’s Rotary Wall Vessel Bioreactor (RWV) and rotary cell cultures of RAW 264.7 osteoclast progenitor cells, Sambandam and associates found that simulated microgravity increased osteoclastogenesis by twofold and upregulated the production/expression of factors involved in osteoclastogenesis, including cytokines, growth factors, proteases, signaling proteins, and transcription factors c-Jun, MITF, and CREB as compared to ground controls [66] (Table 2).

Using the same culture system, Sambandam and associates studied the effects of simulated microgravity on osteoclast precursor autophagy. RAW 264.7 osteoclast precursors cells were cultured for 24 h, and their RNA was extracted and analyzed for the expression of autophagic genes. In addition, marrow cells extracted from the long bones of C57BL/6 mice were cultured for 7 days with added RANKL and M-CSF ± the autophagic inhibitor, 3-methyladenine (3-MA). They found that microgravity increased the expression of autophagic genes and produced multiple autophagosomes in RAW 264.7 cultures. They also found that 3-MA inhibition of autophagy in the RANKL/CSF+ marrow cultures inhibited osteoclastogenesis. They concluded that “autophagy plays an important role in enhanced osteoclast differentiation and could be a potential therapeutic target to prevent bone loss in astronauts during space flight missions” [67].

In a separate study using the same culture system and 24-h cultures of RAW 264.7 osteoclast precursors, Sambandam et. al. found that the expression of tumor necrosis factor-related apoptosis-inducing ligand (TRAIL), TRAF-6, and the fusion genes *OC-STAMP* and *DC-STAMP* were upregulated in simulated microgravity and concluded that “inhibition of TRAIL expression could be an effective countermeasure for µXg-induced bone loss” [68].

Using the RWV, Ethiraj and associates cultured bone marrow cells flushed from the long bones of 6–8-week-old C57BL/6 mice for 24 h, and then subcultured the cells for 7 days with added RANKL and M-CSF ± a syncytin-A knockdown small-interfering RNA (siRNA) and stained the cells for TRAP and syncytin-A. They found that inhibition of syncytin-A decreased microgravity-induced osteoclastogenesis. They concluded that increased syncytin-A production in simulated microgravity upregulated osteoclastogenesis and that “targeting syncytin-A expression may be an effective countermeasure to control bone loss under microgravity conditions” [69] (Table 2, Figure 7).

## 9. Discussion

Unloading of bone on Earth and in the microgravity of space is associated with decreased bone formation and increased bone resorption. The reasons are complex but include the reduction of bone-loading signals normally transduced by shear stresses and hydraulic pressures exerted on osteocytes residing in the lacunar–canalicular network of bone, the increased secretion of sclerostin by pre-apoptotic osteocytes, and microgravity-induced disruption of osteoblast nuclei, cytoskeleton, and intracellular adhesins.

Experiments have been consistent in showing that conditions of microgravity and simulated microgravity decrease the mineral content and cortical and trabecular microstructures of bone, increase osteoclast secretion of sclerostin, decrease osteoblastogenesis and osteoblast secretion of OPG, and increase osteoclast differentiation, fusion, and expression of regulatory and osteoclast-specific genes [57,58,59,60,61,62,63,64,65,66,67,68,69]. However, why, in contrast, to osteocytes and osteoblasts, do osteoclasts with their complex cytoskeletons, remain functional under conditions of bone unloading—both on Earth and in space? How is the integrity of the sealing zone, so essential for bone resorption, maintained under such adverse conditions? What happens to the complex associations of actin and intermediate filaments, septins, and microtubules in osteoclasts subjected to microgravity? Why are M-CSF, RANKL, and calcineurin transcriptional pathways upregulated in osteoclast hematopoietic stems cells but downregulated in osteoblast mesenchymal stem cell precursors in zero gravity? Moreover, is there any relation between osteoclast survival and space-related changes in osteoclast regulation by immune cells and their cytokines? These intriguing questions should provide an ample basis for future research into the amazingly resilient osteoclast, including the development of agents capable of disabling key elements in its cytoskeleton.

In addition to bone unloading, a number of physiopathological conditions are characterized by excessive osteoclast activity. These include but are not limited to menopause, juvenile Paget’s disease of bone, inflammatory joint diseases, bone cancers such as multiple myeloma, and glucocorticoid therapy [1,2]. Thus, it is not surprising that many of the studies on bone homeostasis have been motivated by the need to find treatments capable of modifying osteoclast activity without inducing osteopetrosis [8]. I have listed below several potential treatments designed for this purpose.

### 9.1. Biphosphonates

Bisphosphonates have long been used with success to control osteoclast-mediated bone disease; these agents are incorporated into the bone matrix and are ingested by bone-resorbing osteoclasts, causing their apoptosis. However, biphosphonates inhibit the stimulatory activity of osteoclasts on osteoblast differentiation and, as a consequence, patients on these drugs suffer from a blockade of *de* novo bone formation [12,70].

### 9.2. Anti-RANKL Antibody

A recently developed human monoclonal antibody against RANKL, denosumab, has been shown to have undesirable side effects and, similar to biphosphonates, adversely affects osteoblastogenesis [71].

### 9.3. Cathepsin K Inhibitor

An inhibitor of cathepsin K, odanacatib, was shown to prevent pathological bone loss while preserving bone formation but failed in clinical phase III trials due to increased risk of stroke [72].

### 9.4. Anti-Sclerostin Antibody

Scientists have developed a humanized monoclonal antibody directed against sclerostin (romosozumab), which is approved for the treatment of osteoporosis. Clinical trials have shown that monthly subcutaneous injections of romosozumab are effective in increasing bone formation and density and decreasing bone resorption—results in keeping with the known effects of sclerostin on bone homeostasis [73]. However, there is some concern about the potential cardiotoxicity of romosozumab, prompting the need for further observations [74]. In addition, there is evidence in experimental animals that sclerostin generated in response to TNF-α and IL-1β improves post-traumatic osteoarthritis by inhibiting the activity of proteolytic enzymes involved in cartilage degradation [75]. Because astronauts experience an increased incidence of post-traumatic osteoarthritis involving their knees, ankles, hips, and shoulders, their use of anti-sclerostin antibody during spaceflight may prove to be a double-edge sword. It is important to note that both TNF-α and IL-1β play an important role in the pathophysiology of osteoarthritis [76].

### 9.5. Osteoprotegerin

Osteoprotegerin-Fc given subcutaneously to mice flown for 12 days in space produced a sustained suppression of bone resorption and, thus, deserves further study [77].

### 9.6. Melatonin

Ikegame and associates reported that melatonin, a well-tolerated and widely available compound, stimulated calcitonin mRNA expression and decreased RANKL mRNA expression in cultured fish scales (a surrogate for bone cultures) during an 11-day space flight aboard the International Space Shuttle. Calcitonin is an osteoclast-inhibiting hormone, and, as previously noted, RANKL binding to RANK is required for osteoclastogenesis. The authors posited that melatonin might prove useful in preventing space-related bone loss and, thus, deserves further evaluation [78].

### 9.7. Insulin-Like Growth Factor-1

Insulin-like growth factor (IGF)-1, which plays a major role in all phases of bone and cartilage growth, has been shown to increase rodent humerus periosteal bone formation by 37% during a 10 day Space Shuttle flight [8]. The potential of IGF-1 and other growth factors such as TGF-β and BMP to regulate bone homeostasis in situations of bone unloading merits further investigation.

## 10. Future Prospects

China, Russia, and the United States have plans to establish colonies on our moon sometime in the 2030s and to use the moon as a way station for eventual trips to Mars. These plans include lunar-orbiting space stations. Thus, it would seem that the future holds ample opportunities to study the effects of varying degrees of microgravity on the remarkable and resilient osteoclast.

## 11. Conclusions

Astronauts are at risk of losing bone mass and damaging joint cartilages despite NASA’s efforts to protect their skeletal system by initiating exercise programs and nutritious diets. Bone loss is the consequence of microgravity-related impairment of osteocyte and osteoblast function and the consequent upregulation of osteoclast-mediated bone resorption. Further research is needed to better understand how osteoclasts are able to function in zero gravity and to develop more effective interventions to prevent osteoclast-mediated bone disease.

## Figures and Tables

**Table 1:** Effect of anti-osteoclastogenic cytokines on hematopoietic stem cells.

Cytokine	M-CSF/RANKL Pathways	Calcineurin Pathway	Apoptotic Pathway	RANK/RANKL/OPG Expression	References
IL-4	Blocks JNK, P38, ERK protein kinase signaling. Inhibits NF-κB, c-Fos, NFATc1 transcription	↓ calcium signaling		↓ RANK, RANKL expression ↑ osteoblast OPG expression	[39,40,41,42]
IL-6 sIL-6R	PGE2-mediated ↓ osteoclastogenesis				[44]
IL-10	Inhibits c-Fos, c-Jun, NFATc1 transcription	Inhibits TREM2 expression			[43]
IL-12	↑ IL-18 inhibition of TNF-α-mediated osteoclastogenesis		↑ Fas/Fas ligand expression		[45,47]
IL-18	↓ osteoblast M-CSF production. Inhibits TNF-α-mediated osteoclastogenesis				[46,47]
IFN-γ	Inhibits TNF-α-mediated osteoclastogenesis		↑ Fas/Fas ligand binding		[49]

IL, interleukin; IFN, interferon; PGE2, prostaglandin E2; TNF, tumor necrosis factor; OPG, osteoprotegerin.

**Table 2:** Effect of microgravity on osteoclast hematopoietic stem cell precursors.

Cell	M-CSF/RANKL Pathways	Calcineurin Pathway	Sclerostin Pathway *
HSC	↑ c-Jun, MITF, CREB [63], TRAIL, TRAF-6, *OC-STAMP, DC-STAMP* [64], syncytin-A [60]	↑ S100AB protein and cytosolic calcium [63]	↑ osteoclastogenesis secondary to ↓ OPG production [53]

* Microgravity-related osteocyte apoptosis increase their secretion of sclerostin [60]. TRAIL, tumor necrosis factor-related apoptosis-inducing ligand.
